# Immediate and Long-Term Effects of an 8-Week Digital Mental Health Intervention on Adults With Poorly Managed Type 2 Diabetes: Protocol for a Randomized Controlled Trial

**DOI:** 10.2196/18578

**Published:** 2020-08-04

**Authors:** Eliane Boucher, Judith T Moskowitz, Gina M Kackloudis, Julia L Stafford, Ian Kwok, Acacia C Parks

**Affiliations:** 1 Happify Health New York, NY United States; 2 Feinberg School of Medicine Northwestern University Chicago, IL United States

**Keywords:** online intervention, diabetes mellitus, type 2, mental health, randomized clinical trial, positive psychology, HbA1c

## Abstract

**Background:**

Diabetes is a leading cause of years of life lost and accounts for approximately one-fourth of health care dollars spent in the United States. Many of these costs are related to poor medication adherence and lack of self-care behaviors and are thus preventable. Depression, which is more prevalent among people with diabetes than in the general population, predicts poorer management of one’s diabetes, whereas positive affect predicts engaging in more positive health behaviors. Consequently, interventions that improve depression and positive affect may also improve diabetes-related outcomes among people with diabetes. Although preliminary research on the impact of such interventions among people with diabetes is promising, these studies focused primarily on in-person interventions, have had small samples, and lack long-term follow-up.

**Objective:**

This study aims to examine the short- and long-term effects of a digital therapeutic platform focused on mental health among adults with poorly managed type 2 diabetes and elevated levels of depression.

**Methods:**

This is a randomized controlled trial in which adults with a type 2 diabetes diagnosis, elevated hemoglobin A_1c_ (HbA_1c_) levels (≧7), and moderate to severe depressive symptoms will be randomly assigned to a positive emotion regulation skills intervention group or a sham digital intervention with only psychoeducational content. The study will take place over 14 months, including the 8-week intervention (or control) delivered via a digital therapeutic platform (Happify Health) and follow-up assessments at 3, 6, and 12 months postintervention. Throughout the intervention and for 1 week at each postintervention follow-up, participants will complete daily assessments of diabetes-related distress, diabetes regimen adherence, and mood. Our primary outcome, HbA_1c_, will be self-reported every 3 months throughout the study. Secondary and exploratory outcomes will be assessed at baseline; at 8 weeks; and at 3, 6, and 12 months postintervention.

**Results:**

Recruitment is expected to begin in June 2020. Participants will begin the study as they are recruited and will finish in waves. The final wave of data collection from the 8-week intervention is expected for winter 2020, with the completion of the 12-month follow-up in winter 2021.

**Conclusions:**

Although previous research suggests that in-person psychological interventions have promising effects on both psychological and physical outcomes among adults with diabetes, digital interventions can be advantageous because they are easily scalable and reduce many barriers that prevent people from seeking treatment. This trial will provide important information about the effects of a digital mental health intervention among adults with type 2 diabetes, assessing both short- and long-term effects of this intervention on HbA1c, depressive symptoms, and other diabetes-specific outcomes. If successful, this may introduce a scalable intervention that would help reduce some of the preventable costs associated with diabetes.

**Trial Registration:**

ClinicalTrials.gov NCT04068805; https://clinicaltrials.gov/ct2/show/NCT04068805.

**International Registered Report Identifier (IRRID):**

PRR1-10.2196/18578

## Introduction

### Prevalence and Costs of Diabetes

Diabetes is one of the most common chronic health conditions worldwide [1] and is considered to be a leading cause of years of life lost [2]. The prevalence of diabetes has also been increasing since the mid-1980s [3]. According to the Centers for Disease Control and Prevention, approximately 9.5% of the American population had diabetes in 2015 [4], most of which were type 2 diabetes diagnoses [4], and approximately 34% of the population met the criteria for prediabetes, suggesting that even more people are at risk of developing type 2 diabetes [4]. By 2030, 4.4% of the global population is estimated to have diabetes [5].

The economic cost associated with diabetes is also increasing because of the increased prevalence and the increased cost per patient [6]. The American Diabetes Association recently estimated the total annual cost of diabetes at US $327 billion when considering both medical costs and reduced productivity [7], and people with diabetes account for 25% of all health care dollars spent in the United States [7]. On an individual level, people with a diabetes diagnosis spend approximately US $16,750 annually for medical expenses, and more than 57% of those expenses are directly related to their diabetes [7]. The projected global costs associated with diabetes are estimated to increase from US $1.32 trillion in 2015 to US $2.12 trillion by 2030 [8].

However, many of these costs are preventable, resulting from poor diabetes management. In 2014, 577,040 diabetes-related hospitalizations in the United States were preventable [9], accounting for US $5.9 billion of the diabetes-related health care costs [10]. Previous research shows that many people with diabetes struggle with medication compliance [11], which puts them at an increased risk for hospitalization and mortality [12]. Similarly, adherence to physician-recommended self-monitoring of blood glucose levels tends to be low, particularly among people with type 2 diabetes [13].

### Diabetes and Mental Health

People living with a chronic illness often report that depression is a major barrier to managing their condition and, worse, often do not seek treatment for their depression due to perceived stigma [14]. This is particularly relevant for people with diabetes, who are at a higher risk for depression than the general population [15]. Although depression does not appear to be directly related to hemoglobin A_1c_ (HbA_1c_), a measure of average blood sugar levels over 3 months, in patients with diabetes [16,17], comorbid depression predicts poorer diet, poorer medication compliance, and greater functional impairment [18], which, in turn, predicts higher rates of diabetes-related preventable hospitalizations [19] and health care costs [18]. In addition, depression predicts higher levels of diabetes-related distress [20]. In turn, diabetes-related distress predicts poorer HbA_1c_ [16], and reducing diabetes-related distress leads to significant improvements in HbA_1c_ level [17].

Conversely, higher levels of positive affect are associated with reduced mortality risk [21] and depression [22,23] among people with diabetes. Positive affect also promotes various health behaviors central to managing diabetes, including higher levels of physical exercise [24] and better diet [25]. Similarly, higher levels of self-compassion predict better behavioral and psychological outcomes among people with diabetes as well as better HbA_1c_ levels [26]. Conceivably, self-compassion may lead to improved diabetes outcomes because it promotes greater well-being and better mental health [27]; however, self-compassion may also lead to more adaptive responses to difficulties patients face with managing their condition and, in turn, promote better diabetes management, including more effective health care use, healthy diet and physical activity, and better management of blood glucose levels [26].

Taken together, these findings suggest that better mental health among people with diabetes is associated with less diabetes-related distress and healthier lifestyles, which, in turn, predict better diabetes-related outcomes. Consequently, psychological interventions targeting mental health may lead people with diabetes to engage in healthier lifestyles, indirectly improving diabetes-related outcomes and reducing associated costs [22,28].

### Impact of Mental Health Interventions on Diabetes

Research on the impact of mental health interventions applied specifically to people living with diabetes remains limited. Mindfulness and positive psychology (PP) interventions, which are effective in reducing depression and increasing well-being in the general population [29], have shown promise in improving depression, diabetes-related distress, and HbA_1c_ levels among people with type 1 or 2 diabetes [30]. A recent systematic review showed that such interventions, particularly mindfulness-based interventions, led to improvements in psychological outcomes, including self-efficacy, self-compassion, well-being, mental health–related quality of life, positive affect, stress, diabetes-related distress, depression, and anxiety [30]. Mindfulness-based interventions also appeared to lead to improvements in HbA_1c_ levels, although in some cases, the effects were not observed until 1 or 3 months postintervention [30]. Other research also suggested that mindfulness-based interventions have small to moderate effects on metabolic control [31].

Such interventions appear to be particularly successful when patients have higher baseline levels of diabetes-related distress and when interventions are delivered in a group format, draw on mindfulness-based stress reduction (MBSR), and include home practice assignments [32]. Although few of these studies have included long-term follow-up, there is preliminary evidence that the effects of mindfulness-based interventions on depressive symptoms are maintained, or even stronger, after 6 months [30,32], although they may dissipate after 2 or 3 years postintervention [30]. Some findings also suggest that the effects of mindfulness-based interventions on HbA_1c_ levels may actually be stronger 1 or 3 months postintervention rather than immediately postintervention [30].

Preliminary research also suggests that cognitive behavioral therapy (CBT) may be effective in treating depression among individuals with diabetes, although the effects on diabetes-specific outcomes are mixed, and may be limited to people with high baseline depression scores [33]. In one study of adults with uncontrolled diabetes and unipolar depression, CBT designed for adherence and depression led to improvements in medication adherence and self-monitoring of blood glucose, HbA_1c_, and depression, and these improvements were maintained for up to 12 months [34]. However, in another study, CBT was associated with improved depression relative to a control group, but the levels of glycosylated hemoglobin were better in the CBT group than in the control group only at 6 months posttreatment [35]. Similarly, a recent systematic review of 10 randomized controlled trials (RCTs) showed that CBT improved depression, quality of life, fasting glucose, and anxiety but not glycemic control or diabetes-related distress [36].

### Digital Interventions

Although this research is promising, most of these studies have focused on the impact of in-person interventions. However, there are numerous barriers to in-person treatment that prevent people from seeking treatment [37], including stigma [38] and a shortage of mental health professionals [39]. Internet-based interventions offer a cost-effective means of providing access to large populations [40], while also reducing some of the other barriers to seeking treatment by increasing convenience [41] and anonymity [42]. Indeed, research suggests that people with comorbid depression and diabetes find web-based interventions attractive and may reach a population that would not otherwise seek treatment [43].

Although previous research supports the effectiveness of internet-based interventions in physical health [44] and mental health [45] domains, few studies have tested the impact of internet-based interventions among people with diabetes specifically, and what research has been done on the benefits of mobile health interventions among people with diabetes yielded mixed results [46]. Most studies on the effects of web-based interventions on people with diabetes found no significant effects on depression or distress [47,48], whereas research demonstrating effects on depressive symptoms found no effects on diabetes-specific outcomes [22]. However, unlike most studies of in-person CBT, these studies did not include a sample of patients with diabetes and elevated levels of depression. One study of individuals with a diabetes diagnosis and elevated depressive symptoms found that internet-based CBT reduced depression, although they found no benefits for glycemic control [49]. Therefore, additional research testing the effectiveness of digital mental health interventions specifically among people with diabetes and elevated depressive symptoms is necessary, particularly studies with long-term follow-up and larger sample sizes [47].

### Objectives of This Study

This study aims to examine the effectiveness of a digital therapeutics platform named *Happify Health* on people with poorly managed type 2 diabetes and elevated depressive symptoms. Happify Health is a digital intervention platform focused on mental health and its impact on other diseases that can be accessed via the internet or mobile app. Unlike most other interventions tested in person or digitally, which draw on just one theoretical approach, Happify Health activities draw from each of the three major theoretical approaches: CBT [50], MBSR [51], and PP [52-54]. Therefore, Happify Health uses multiple pathways to improve users’ mental health by incorporating strengths from all three theoretical approaches, while simultaneously improving person-activity fit relative to other singular approaches by offering users more opportunities to find types of activities that suit them.

Activities within Happify Health were developed by identifying evidence-based tasks and interventions that were shown to be effective in at least two separate studies and in different samples [55]. These activities fall into different themes: *savor* (building mindfulness skills), *thank* (gratitude), *aspire* (optimism, goal setting, and finding meaning and purpose), *give* (kindness, forgiveness, and prosociality), *empathize* (self-compassion and perspective taking), and *revive* (physical health). Activities from these different themes are then organized into *tracks*, developed to help users improve in a specific area of concern such as reducing stress. Within each track, users can select specific activities and may also switch tracks before completing them or access activities in a *free play* format. Consequently, users have the ability to choose tracks and activities that better fit them, which has been shown to improve outcomes [56].

Observational studies of Happify Health users demonstrate that usage over 8 weeks is associated with more than a 27% increase in positive emotions, and high-use participants see even greater improvement [57,58]. A later RCT also demonstrated that Happify Health users who completed at least two activities per week reported significantly more improvement in depression, anxiety, and resilience than those who completed fewer activities or participants assigned to the control group [59]. Similar effects were obtained from RCTs focused specifically on people with higher levels of emotional or workplace distress [60]. More recent research also suggests that Happify Health use improves subjective well-being among people living with chronic physical conditions, including, but not limited to, diabetes, at the same rate as those without chronic conditions [61]. Consequently, there is preliminary evidence to suggest that Happify Health could effectively improve subjective and psychological well-being among individuals diagnosed with type 2 diabetes. However, no research has examined whether Happify Health use could also positively improve outcomes associated with their diabetes, including how well they manage their condition, their level of diabetes-related distress, and physical outcomes such as HbA_1c_ levels.

Therefore, in this study, our goal is to examine whether Happify Health use over the course of 8 weeks also helps to improve diabetes-specific outcomes. To do so, we plan to compare changes in HbA_1c_ levels among adults with poorly managed type 2 diabetes and elevated depressive symptoms who have completed 8 weeks of activities on Happify Health or 8 weeks of a sham digital intervention. Secondary outcomes include depression, positive affect, and other diabetes-specific outcomes such as medication adherence, diabetes-related distress, and diabetes-related self-care activities. In addition, although many other studies (particularly mindfulness and PP interventions) did not examine long-term effects, we plan to explore the long-term effects of Happify Health use on both primary and secondary outcomes at 3, 6, and 12 months postintervention.

## Methods

### Recruitment Strategy

We plan to recruit participants by capitalizing on the existing process used to draw new users to Happify Health, including advertisements on Facebook and other social media sources. To attract individuals with type 2 diabetes specifically, targeted advertisements will also be posted on websites that connect potential participants with research studies and clinical trials (eg, Research Match) and on websites relevant to people living with type 2 diabetes.

Interested participants will be directed to a web-based survey to determine eligibility. The survey questions will include questions on age, location, previous Happify Health usage, diagnosed chronic illnesses, self-reported HbA_1c_ level, the Patient Health Questionnaire (PHQ) [62], and contact information. Users who meet initial inclusion criteria will then be contacted directly by Happify Health research staff to discuss the study in more detail. During this call, all eligibility questions will be readministered to ensure that the potential participant still meets the inclusion criteria. Those who agree to participate after this call will be sent an email to begin a week-long run-in period; during this run-in period, participants will be instructed to answer a brief web-based survey including three questions assessing daily diabetes-related distress, diabetes regimen adherence, and mood. Participants who complete this run-in period will then be sent an email instructing them to download the Happify Health app and to begin the study.

### Inclusion and Exclusion Criteria

Participants will qualify for the study if they are aged 18 years or older, currently living in the United States, have never used Happify Health before, and have a current diagnosis of type 2 diabetes. In addition, as we are targeting participants with poorly managed diabetes and recent recommendations are that below 7% is a reasonable glycemic goal for most adults with type 2 diabetes [63], participants will be included if their most recent HbA_1c_ level is at least 7%. Similarly, as we are also targeting participants with elevated depressive symptoms, participants will be included if their scores on the PHQ are at least 15, which is indicative of moderately severe depression [62]. Participants will also need to indicate their willingness to provide HbA_1c_ results throughout the study, complete daily assessments, and engage with the platform to qualify for the study. Finally, participants will also have to complete 5 of the 7 daily questionnaires during an initial qualifying period (refer to the *Procedures* section for more detail) to participate in the study.

### Participants

This study is an RCT (NCT04068805) with an initial target sample size of 400 participants (200 participants per condition). Recruitment will continue until 400 participants have successfully completed pretesting and have been randomized to condition.

Although previous RCTs using Happify Health had response rates ranging from 56% to 72% for an 8-week posttest [60], as the first attempt to include long-term follow-up of Happify Health users, the level of attrition beyond the 8-week intervention is difficult to estimate. The few previous tests of mental health interventions on people with diabetes that included long-term follow-up reported relatively low attrition for 6-month [35,64] and 12-month [34,65] follow-ups; however, all these studies involved in-person interventions and often included booster sessions, thereby increasing the level of contact with participants and increasing response rates. The levels of attrition are much higher in research using digital interventions [66], although dropout rates are lower in RCTs than in open-access interventions [67]. Consequently, although we predict attrition for long-term follow-ups to be higher in this study than in previous research with in-person interventions, it is unclear what percentage of participants to expect for our 3-, 6-, and 12-month assessments.

### Participant Compensation

Participants will receive three types of compensation throughout the study. After the baseline assessment (for which participants will not be compensated), participants will be compensated with Amazon gift cards valued at US $15 for completing each assessment. As separate compensation for completing daily assessments (as part of the 8-week intervention and during the 3-, 6-, and 12-month follow-ups), participants will earn US $1 for each daily assessment, for a possible total of US $56 for completing daily assessments during the intervention and US $28 for completing the week-long daily assessments at postintervention and during the 3-, 6-, and 12-month follow-ups. Finally, participants will be compensated with US $5 for obtaining and reporting each HbA_1c_ recording after baseline. Thus, participants will be compensated with a total of US $164 if they complete all assessments at all waves of data collection.

### Outcome Measures

All assessments will be administered via Happify Health, and participants who do not complete measures will be sent email reminders; however, participants may withdraw from the study or skip assessments at any time. To link participant data across assessments and other data collections, each participant will be assigned a unique study ID number; participants will otherwise remain anonymous throughout the study. In addition to the primary and secondary outcome measures described in the following sections, other exploratory measures will also be included that are not reported here.

### Primary Outcome: Hemoglobin A_1c_

Participants will self-report their HbA_1c_ levels, a measure of the average blood glucose levels over the past 3 months, 5 times throughout the study: at baseline, week 12 (3 weeks postintervention), week 24 (3 weeks after the 3-month postintervention assessment), week 36 (3 weeks after the 6-month postintervention assessment), and week 57 (at the 12-month postintervention assessment). At each assessment, participants will also report the date when they received this HbA_1c_ reading to verify that it falls in the correct time frame.

### Secondary Outcomes

#### Diabetes Distress Scale

Diabetes distress scale [68] is a 17-item scale that assesses the extent to which certain diabetes-related situations have been a problem for participants. Each item represents a potential problem area for individuals living with diabetes (eg, feeling that I am often failing with my diabetes routine), and participants indicate the extent to which they have been distressed or bothered by that issue during the past month on a scale ranging from 1 (*not a problem*) to 6 (*a very serious problem*). The responses are averaged so that scores can range from 1 to 6, where higher scores indicate higher levels of distress, and scores of 3 or greater are considered to be clinically significant levels of diabetes-related distress.

#### Measures of Medication Adherence Scale

Measures of Medication Adherence Scale [69] is a 4-item self-report measure of medication adherence and thus will only be shown to participants who indicate they are currently prescribed medication for diabetes on a preceding question. This scale will be modified slightly to ask participants specifically about adherence to diabetes medication over the past week. For each item (eg, Over the past week, did you ever forget to take your diabetes medicine?), participants indicate *yes* or *no*. All *yes* responses are coded as 0, and *no* responses are coded as *1*. The responses are then summed so that higher scores indicate greater medication nonadherence.

#### Summary of Diabetes Self-Care Activities

Summary of diabetes self-care activities [70] is an 11-item measure of self-reported adherence to diabetes self-care activities. For 10 items, participants indicate on how many days they engaged in self-care activities related to diet (eg, How many of the last 7 days have you followed a healthful eating plan?), exercise (eg, On how many of the last 7 days did you participate in at least 30 min of physical activity?), blood sugar testing (eg, On how many of the last 7 days did you test your blood sugar?), and foot care (eg, On how many of the last 7 days did you check your feet?) over the past week on a scale ranging from 0 to 7. One item also asks participants to indicate the frequency of smoking over the past week (ie, Have you smoked a cigarette, even 1 puff, during the past 7 days?) on a scale ranging from 0 (*no*) to 1 (*yes*); participants who respond yes are then asked to indicate how many cigarettes they smoked on an average day. Separate scores are then created for each subscale.

#### Patient Health Questionnaire

PHQ-9 [62] is a 9-item scale measuring depressive symptomology. Each item represents a depressive symptom (eg, feeling down, depressed, or hopeless), and participants indicate how often they have been bothered by each symptom over the past 2 weeks on a scale ranging from 0 (*not at all*) to 3 (*nearly every day*). The responses are then summed such that scores can range from 0 to 27, and higher scores indicate more severe depressive symptoms.

#### Patient-Reported Outcomes Measurement Information Systems: Positive Affect Subscale

Participants will also complete the positive affect subscale of the Patient-Reported Outcomes Measurement Information Systems [71] scale. This subscale asks participants to indicate how much they felt each of the 15 emotions (eg, “I felt cheerful”) over the past 7 days on a scale ranging from 1 (*not at all*) to 5 (*very much*). Items are summed so that higher scores indicate greater positive affect.

### Daily Assessments

#### Diabetes-Related Distress

We will assess daily levels of diabetes-related distress with a single item (ie, How would you rate your diabetes-related distress, on average, over the past 24 hours?) rated on a scale ranging from 1 (*very low*) to 7 (*very high*).

#### Diabetes Regimen Adherence

We will assess participants’ daily regimen adherence using a single item (ie, Which of the following activities in your diabetes regimen did you complete over the past 24 hours?) where participants check any of the following options that apply to their situation: monitored blood sugar, ate according to healthy eating diet, engaged in physical activity, took diabetes medication as prescribed, or did not follow my diabetes regimen at all.

#### Daily Mood

We will assess participants’ daily mood with a single item (ie, “How depressed did you feel, on average, over the past 24 hours?”) rated on a scale ranging from 1 (*not at all*) to 7 (*extremely*) [72].

### Procedures

Participation in this study will take place over approximately 14 months, including an 8-week intervention (or corresponding control) delivered via Happify Health as well as follow-up assessments at 3, 6, and 12 months postintervention. To ensure that HbA_1c_ levels reported postintervention reflect average glucose levels only after starting the intervention, all participants will begin the study within 3 weeks of obtaining their most recent HbA_1c_ levels. Participants who indicate that their most recent HbA_1c_ levels were obtained before that when contacted by phone will wait to begin the study until they obtain their following HbA_1c_ measurement and researchers confirm that they meet the inclusion criterion.

Once participants are ready to begin the study, they will be directed to complete 1 week of daily assessments without exposure to either condition. Participants who complete fewer than 5 of these assessments will be disqualified from the study. Those who complete at least five of the daily assessments during this qualifying period will be directed to download the mobile app and complete the regular onboarding questionnaire for Happify Health; participants will be randomly assigned to either the positive emotion regulation skills intervention group or the control group upon completing these questions. Following randomization, participants will be prompted to complete the baseline assessment.

Participants will then be instructed to begin using their assigned version of Happify Health. Although participants will not be given explicit instructions on how often they should use the platform, they will be encouraged to engage with the platform daily. Participants will receive daily push notifications on their mobile device to remind them to access the platform and answer daily questionnaires, and they will also receive weekly emails as part of the Happify Health platform to help keep them engaged with the program. In addition, to improve use rates and participant retention, research staff will call participants to inquire about problems when a participant has not engaged with the Happify Health platform at all for 1 week.

After 8 weeks, participants will be instructed to complete the postintervention assessment; this assessment will be identical to the baseline with the exception of HbA_1c_ assessment, which will occur 3 weeks later via the Happify Health app. After completing this assessment, participants will receive an email instructing them to answer daily questions for 1 week and to remind them that we will contact them again in 3, 6, and 12 months for follow-up assessments and that they should continue to use their assigned version of Happify Health as they see fit. Participants will then receive emails instructing them to complete each of the 3-, 6-, and 12-month assessments, which will also be accompanied by 1 week of daily assessments at each follow-up period. Table 1 provides the schedule of activities. To improve participant retention, participants who do not complete the postintervention, 3-, 6-, and 12-month assessments will also receive a reminder phone call from the research staff.

**Table 1:** Schedule of activities for prescreen, intervention period, and follow-up assessments.

Assessments		Time										
		Prescreen	Week 1: qualifying period	Week 2: baseline and first week of intervention	Weeks 3-9: intervention	Week 10	Week 12	Week 21: 3-month follow-up	Week 24	Week 33: 6-month follow-up	Week 36	Week 57: 12-month follow-up
**Primary outcome**												
	HbA_1c_^a^	X^b^	N/A^c^	X	N/A	NA/	X	N/A	X	N/A	X	X
**Secondary outcomes**												
	Diabetes distress scale	N/A	N/A	X	N/A	X	N/A	X	N/A	X	N/A	X
	Measures of Medication Adherence Scale	N/A	N/A	X	N/A	X	N/A	X	N/A	X	N/A	X
	Summary of diabetes self-care activities	N/A	N/A	X	N/A	X	N/A	X	N/A	X	N/A	X
	Patient Health Questionnaire	X	N/A	X	N/A	X	N/A	X	N/A	X	N/A	X
	Positive affect subscale	N/A	N/A	X	N/A	X	N/A	X	N/A	X	N/A	X
	Daily assessments	N/A	X	X	X	X	N/A	X	N/A	X	N/A	X

^a^HbA_1C_: hemoglobin A_1c_.

^b^X: assessment administered.

^c^N/A: not applicable.

### Intervention Group

**Figure 1:**
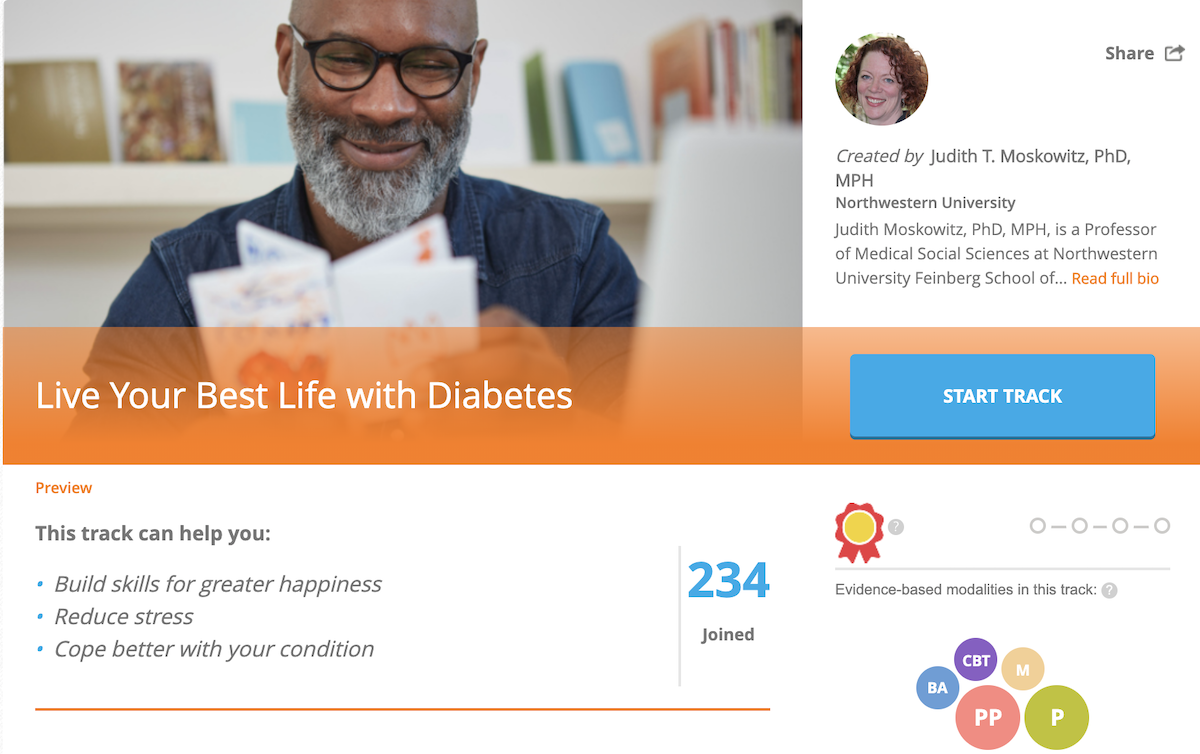
Screenshot of the featured track description for Developing Affective HeaLth to Improve Adherence intervention.

Participants assigned to the positive emotion regulation skills intervention will receive full access to the Happify Health platform. However, their version will feature a diabetes-related track focusing on building skills for greater happiness, reducing stress, and coping better with diabetes (Figure 1). Although the description of the track cues them to think about the impact of negative emotions on their diabetes and the benefits of increasing positive emotions, activities within the track are not focused specifically on diabetes. Importantly, although participants will be encouraged to use this particular track, they will not be required to start or complete the diabetes-related track and will be able to choose from all the other tracks available on Happify Health.

The featured diabetes-related track was designed by Happify Health and the second author, who is an expert on emotion and diabetes. The track is based on activities and content from the Developing Affective Health to Improve Adherence intervention originally developed by Moskowitz et al [21]. The version included in this study has four parts.

#### Part 1

This part contains 8 different activities and exercises focusing on savoring. For example, in 1 activity, *Today’ s grateful moment*, participants are asked to spend a few moments writing about something they are grateful for. In another activity, *Let the good times roll*, participants make a plan to spend time doing simple things that promote positive affect and then write about how it went after completing the tasks they planned.

#### Part 2

This part contains 9 different activities and exercises focusing on reframing negative thoughts. For example, in *A week’s worth of thanks*, participants keep a gratitude journal about someone they are close to and spend time writing about why they are grateful for that person. In another activity, *Savor the small stuff*, participants spend a week doing something mindfully (eg, eating a meal or taking medication) and then write about how it went.

#### Part 3

This part contains 11 different activities that build on parts 1 and 2 but includes exercises promoting goal orientation and optimistic thinking. For example, in *Give Myself a Break*, where participants practice self-compassion and then spend time writing about how it went. In another activity, *Loving-Kindness Meditation*, participants verbalize good wishes when interacting with other people and then write about how it went.

#### Part 4

This part contains 11 different activities that promote resilience and helping others. For example, in *What am I proud of?*, participants spend some time thinking about something they are proud of and then write about it. In another activity, *Help Someone*, participants spend some time helping another person and then write about it.

Participants must complete one part before they can begin the following part, and they do so by earning either a silver medal (earned by completing all but 4 activities) or a gold medal (earned by completing all but 3 activities). Thus, participants will take varying lengths of time to complete a track, depending on their level of engagement with the platform. As participants will have access to the full Happify Health platform, if they complete the intervention track before the 8-week study period has elapsed or choose to change tracks before finishing, they will have access to all other available Happify Health tracks and instant-play activities (where they can choose to complete certain activities outside of dedicated tracks).

### Sham Digital Intervention

**Figure 2:**
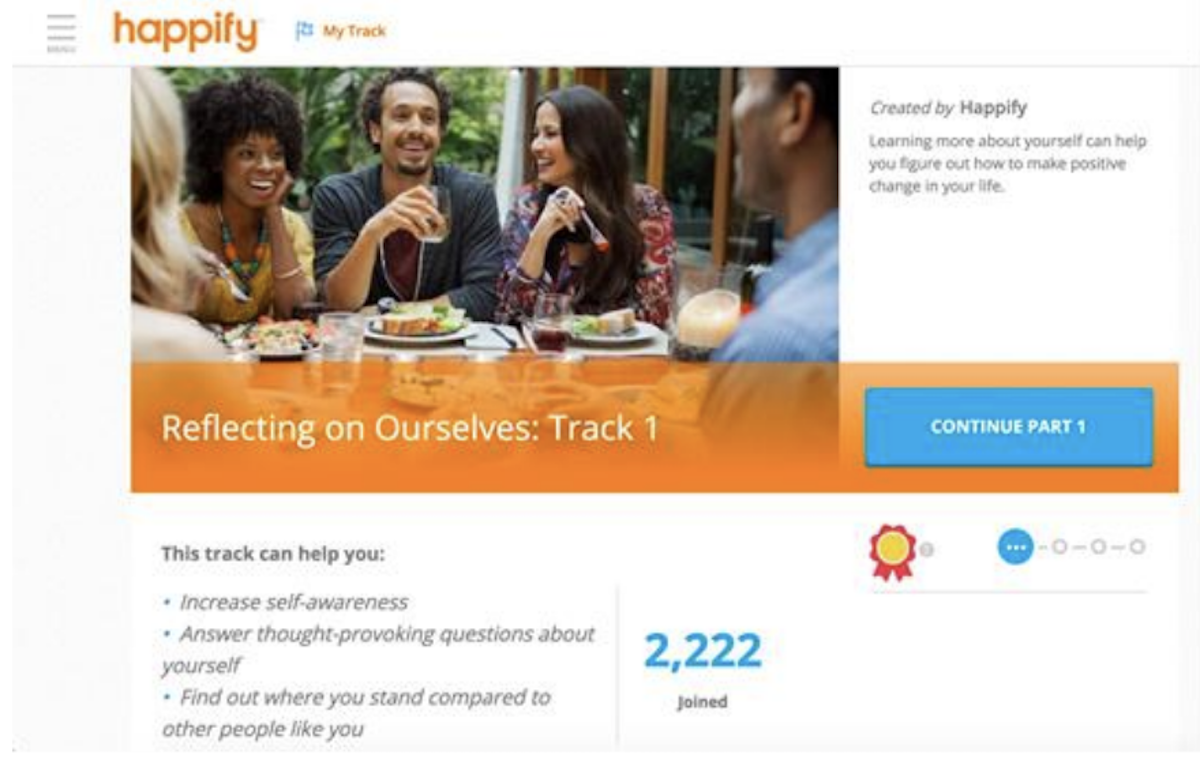
Screenshot of the track description in sham digital intervention.

Participants assigned to the control condition (Figure 2) will have access to a version of Happify Health that includes only polls on various mental health topics. After each poll, participants in this condition are provided with social comparison data about how their responses to the poll compared with other users’ responses and information about why this topic is important (including references to relevant scientific studies). For example, after responding to a poll question that asks participants to indicate how often they have a deep or meaningful conversation with someone, participants will view psychoeducational information about the benefits of conversation, including that people who spend less time alone and more time talking to others tend to be happier [73] and that the happiest people have much less small talk, but many more meaningful conversations compared with the least happy people [74].

### Analysis Plan

We plan to analyze the changes in primary and secondary outcomes across the 2 conditions using hierarchical linear modeling (HLM). We will compute the change trajectories on each outcome over the course of the study assessments (ie, at baseline, immediately postintervention, and at 3-, 6-, and 12-month follow-ups). These trajectories will then be averaged together within each condition, so we can compare the trajectories for each outcome across the 2 conditions to determine if there was greater improvement in the positive emotion regulation skills intervention group relative to the control group.

We plan to use participants’ daily assessments in 2 ways. First, we will compute growth trajectories for each participant using their daily assessments to explore when people tend to experience improvements in diabetes-related distress, diabetes regimen adherence, and mood during the intervention. Second, as retrospective reports can sometimes lead to inflated or inaccurate information compared with daily assessments [75], we will use daily assessments to compute weekly averages for diabetes-related distress, diabetes regimen adherence, and mood during and after the intervention. These weekly averages will then be used as outcome variables in the same HLM analyses described earlier.

Previous research on the impact of health care interventions on people with poorly managed diabetes suggests that effects may be most effective among people with especially poor glycemic control or HbA_1c_ levels above 9.5% [76]. Consequently, we plan to examine the effects of baseline HbA_1c_ as a moderator of the effects of the intervention on changes in the primary and secondary outcomes. Similarly, we also plan to examine the effects of baseline depressive symptoms as a moderator of the effects of the intervention. Finally, given that the effects of Happify Health use are typically moderated by usage [57-61], we plan to examine the effects of frequency of usage as another moderator of changes in the primary and secondary outcomes.

### Ethics

This study was submitted and approved for ethical review by IntegReview (protocol HLS-07), an independent institutional review board.

## Results

Recruitment for the trial is expected to begin during the second quarter of 2020, and participants will begin the 8-week intervention as they are recruited and consent to participate in the study. Consequently, the first wave of data collection is expected to be complete approximately 8 weeks after recruitment, with an estimated intervention completion date for all participants in the fourth quarter. We plan to conduct data analysis for postintervention assessments once this initial data collection is complete, and these results may be shared at conferences or submitted for publication while we continue to collect data for the 3-, 6-, and 12-month follow-up assessments. Owing to the high cost associated with this research, we plan to conduct preliminary analyses once 20% of our target sample has completed the 8-week intervention. If we find no trends suggesting Happify Health use predicts primary outcomes, we plan to discontinue the study.

Recruitment, data monitoring for participant safety, data storage, protocol implementation, and research administration (participant follow-up, etc) will be under the purview of EB, GK, JS, and AP as employees of Happify Health. All data analyses will be conducted by JM and IK.

## Discussion

Previous research suggests that many of the costs associated with diabetes are preventable when individuals with a diabetes diagnosis manage their condition more effectively [9,10,12]. One factor that appears to interfere with individuals’ ability to manage their diabetes is depression [18], which is more common among people with a diabetes diagnosis than in the general population [15]. Interventions targeting depression among people with diabetes show promise in helping to improve psychological as well as physical health outcomes [30,31]. Research testing these interventions, however, focuses primarily on in-person interventions and has yielded mixed results, particularly on diabetes-specific outcomes. The purpose of the outlined study is to examine the effectiveness of a digital therapeutics platform (Happify Health) shown to improve mental health over the course of 8 weeks among general populations [59], distressed populations [62], and people living with chronic physical conditions [64], specifically in a population of adults with poorly controlled type 2 diabetes and elevated depressive symptoms.

### Strengths and Limitations

Previous research demonstrated that 8 weeks of Happify Health use improved subjective well-being among people with chronic physical conditions, including type 2 diabetes, at the same rate as people without these conditions [61]. However, the previous study examined the effects of Happify Health use on users with at least one self-reported chronic condition but was unable to examine the impact on users with specific chronic conditions. Therefore, this will be the first attempt to examine the effects of Happify Health use specifically on individuals with type 2 diabetes. Moreover, the previous study assessed only well-being, whereas this study will examine depressive symptoms and, more importantly, diabetes-specific outcomes such as HbA_1c_, frequency of self-care behaviors, and diabetes-related distress. Consequently, this study will provide a more comprehensive analysis of whether Happify Health use can improve physical health outcomes as well as psychological outcomes among individuals with poorly managed type 2 diabetes and elevated depressive symptoms.

This study also plans to include 3-, 6-, and 12-month follow-up assessments, providing important information about the longitudinal effects of this intervention. Longitudinal research on the impact of psychological interventions on people with diabetes remains limited [30], but research suggests that long-term follow-ups are important, as some effects may dissipate over time [30], whereas other effects may only emerge or become stronger over time [30,31].

Previous research has also been characterized by small samples, with sample sizes for in-person interventions ranging from 23 to 139 [30]. Sample sizes for research testing digital interventions have often been higher; however, most of these studies assessed only psychological well-being (depression and diabetes-related distress) and not physical health outcomes such as glycemic control [47]. Although the levels of attrition for long-term follow-up assessments are difficult to predict, based on response rates in previous RCT research using Happify Health ranging from 56% to 72% for 8-week interventions [60], our sample size postintervention is likely to be substantially higher than that in the previous studies. Given the mixed findings with glycemic control in previous research, it is important to examine these effects with larger samples to determine whether previous null findings are related to a lack of power.

However, one limitation of this study is that we are targeting a specific group of people with poorly managed diabetes and elevated depressive symptoms. Furthermore, because participants will have to successfully complete a run-in period before starting the study, our participants are also likely to differ from those who do not participate in terms of motivation, conscientiousness, etc. Thus, it is unclear whether our findings will generalize to a broader population of individuals with type 2 diabetes.

Another limitation of this study is that the intervention does not specifically focus on diabetes-related content. That is, participants in the positive emotion regulation skills intervention group may choose to complete the featured track, which includes a description cueing them to think about the impact of negative emotions on their diabetes, but activities within this featured track do not refer specifically to diabetes or incorporate diabetes-specific behavioral strategies. Furthermore, participants are not required to complete, or even begin, this track and may choose from any other available tracks that do not refer to diabetes whatsoever. Consequently, our intervention is similar to mindfulness or PP interventions that are broader in focus [30]. By comparison, CBT applied to people with diabetes typically has been modified to include specific information about adherence [34] or include supportive diabetes information [35]. Thus, our intervention may have weaker effects, particularly on diabetes-specific outcomes, than CBT modified for diabetes. However, there is preliminary evidence that completing regular activities (ie, not disease specific) on Happify Health is associated with improved subjective well-being among people with chronic physical conditions [61] and that mindfulness and PP interventions without behavioral strategies for managing diabetes can improve participants’ health outcomes [30]. If we are able to demonstrate that Happify Health use can have physical as well as psychological benefits for people with poorly managed type 2 diabetes and elevated depressive symptoms, without incorporating specific behavioral strategies for managing diabetes, the effects are also likely to have broader applicability.

### Conclusions

Given the buffering effects of psychological well-being on diabetes outcomes [23], researchers and practitioners have become increasingly interested in whether psychological interventions can improve diabetes-related outcomes among people living with diabetes [30]. This trial would add to the emerging literature testing the effectiveness of such interventions. Importantly, however, although most previous research focused on interventions delivered in person [30], this study tests the efficacy of a digital intervention, which has the added benefit of reducing many of the barriers that prevent people from seeking treatment [77]. This study also addresses two important limitations of previous research: (1) small sample sizes and (2) lack of long-term follow-ups [30]. Consequently, this trial will not only provide information about the immediate effects of a digital therapeutic intervention on diabetes-related outcomes but also the extent to which these effects may persist for up to 12 months.

## Abbreviations

CBT: cognitive behavioral therapy
HbA_1c_: hemoglobin A_1c_
HLM: hierarchical linear modeling
MBSR: mindfulness-based stress reduction
PHQ: Patient Health Questionnaire
PP: positive psychology
RCT: randomized controlled trial
